# How Can Biomolecules Improve Mucoadhesion of Oral Insulin? A Comprehensive Insight using Ex-Vivo, In Silico, and In Vivo Models

**DOI:** 10.3390/biom10050675

**Published:** 2020-04-27

**Authors:** Mariana Amaral, Ana Sofia Martins, José Catarino, Pedro Faísca, Pradeep Kumar, João F. Pinto, Rui Pinto, Isabel Correia, Lia Ascensão, Ricardo A. Afonso, M. Manuela Gaspar, Adília J. Charmier, Isabel Vitória Figueiredo, Catarina Pinto Reis

**Affiliations:** 1 Research Institute for Medicines (iMed.ULisboa), Faculty of Pharmacy, Universidade de Lisboa, 1649-003 Lisboa, Portugal; marianaamaral8b@gmail.com (M.A.); anamartins6@campus.ul.pt (A.S.M.); jfpinto@ff.ulisboa.pt (J.F.P.); rapinto@ff.ulisboa.pt (R.P.); mgaspar@ff.ulisboa.pt (M.M.G.); 2 Faculdade de Medicina Veterinária, Universidade Lusófona de Humanidades e Tecnologias/DNAtech Laboratório Veterinário, 1749-024 Lisboa, Portugal; jcatarino93@hotmail.com (J.C.); pedrofaisca76@gmail.com (P.F.); 3 Department of Pharmacy and Pharmacology, School of Therapeutic Sciences, Faculty of Health Sciences, University of the Witwatersrand, Johannesburg 2193, South Africa; pradeep.kumar@wits.ac.za; 4 JCS. Dr. Joaquim Chaves, Laboratório de Análises Clínicas, 1495-068 Miraflores-Algés, Portugal; 5 Centro de Química Estrutural, Departamento de Engenharia Química, Instituto Superior Técnico, Universidade de Lisboa, Av. Rovisco Pais, 1049-001 Lisboa, Portugal; icorreia@tecnico.ulisboa.pt; 6 Centro de Estudos do Ambiente e do Mar (CESAM), Faculdade de Ciências, Universidade de Lisboa, Campo Grande, 1749-016 Lisboa, Portugal; lmpsousa@fc.ul.pt; 7 CEDOC, NOVA Medical School/Faculdade de Ciências Médicas (NMS/FCM), Universidade Nova de Lisboa, 1150-082 Lisboa, Portugal; ricardo.afonso@nms.unl.pt; 8 Ciências Funcionais e Alvos Terapêuticos, NOVA Medical School, Faculdade de Ciências Médicas (NMS|FCM), Universidade Nova de Lisboa, 1169-056 Lisboa, Portugal; 9 Departamento de Física, Faculdade de Ciências e Tecnologia, Universidade Nova de Lisboa, 2829-516 Caparica, Portugal; 10 DREAMS, Universidade Lusófona de Humanidades e Tecnologias, Campo Grande 376, 1749-024 Lisboa, Portugal; januarioadilia@gmail.com; 11 Pharmacology & Pharmaceutical Care, Faculty of Pharmacy, Universidade de Coimbra, 3000-548 Coimbra, Portugal; isabel.vitoria@netcabo.pt; 12 Coimbra Institute for Clinical and Biomedical Research (iCBR), University de Coimbra, 3000-370 Coimbra, Portugal; 13 IBEB, Biophysics & Biomedical Engineering, Faculdade de Ciências, Universidade de Lisboa, 1749-016 Lisboa, Portugal

**Keywords:** marine-derived biomolecules, diabetes mellitus, insulin, mucoadhesion, nanoparticle, oral delivery

## Abstract

Currently, insulin can only be administered through the subcutaneous route. Due to the flaws associated with this route, it is of interest to orally deliver this drug. However, insulin delivered orally has several barriers to overcome as it is degraded by the stomach’s low pH, enzymatic content, and poor absorption in the gastrointestinal tract. Polymers with marine source like chitosan are commonly used in nanotechnology and drug delivery due to their biocompatibility and special features. This work focuses on the preparation and characterization of mucoadhesive insulin-loaded polymeric nanoparticles. Results showed a suitable mean size for oral administration (<600 nm by dynamic laser scattering), spherical shape, encapsulation efficiency (59.8%), and high recovery yield (80.6%). Circular dichroism spectroscopy demonstrated that protein retained its secondary structure after encapsulation. Moreover, the mucoadhesive potential of the nanoparticles was assessed in silico and the results, corroborated with ex-vivo experiments, showed that using chitosan strongly increases mucoadhesion. Besides, in vitro and in vivo safety assessment of the final formulation were performed, showing no toxicity. Lastly, the insulin-loaded nanoparticles were effective in reducing diabetic rats’ glycemia. Overall, the coating of insulin-loaded nanoparticles with chitosan represents a potentially safe and promising approach to protect insulin and enhance peroral delivery.

## 1. Introduction

Diabetes mellitus is a group of metabolic disorders that arise from defective action and/or secretion of insulin, resulting in hyperglycemia [1]. Type 1 diabetes is characterized by the life-long need of exogenous insulin replacement. These patients have the need to self-administer long-acting insulin in order to establish basal levels, and short-acting insulin before meals [2,3]. Insulin administration is done by subcutaneous injection or by constant subcutaneous infusions [3]. Although this route is considered the only option for insulin therapy, and very efficient and broadly used, there are drawbacks and disadvantages associated with its usage [4,5]. Some of these include lipoatrophy and lipohypertrophy on the injection site and discomfort [6]. In addition, all tissues are being exposed to equal quantities of insulin [7], being that insulin reaches the muscles and adipocytes prior to the liver [8], with only around 20% of the administered insulin reaching this target organ [9]. The peripheral hyperglycemia might lead to unwanted overstimulation of the metabolic responses [6]. When insulin is administered subcutaneously, it is first distributed to the peripheral tissues [10]. Thus, this route of administration does not mimic the endogenous insulin produced by the islets of Langerhans by non-diabetics. When administered orally, exogenous insulin is absorbed in the intestine, reaching the liver through the portal vein and inhibiting hepatic glucose output, mimicking the physiological pathway by undergoing hepatic first passage [10,11]. Although oral delivery of insulin is the most comfortable and convenient route for the patient, it also has difficulties associated with its usage due to its protein nature [12]. Such difficulties include poor absorption by the intestine epithelium due to insulin’s hydrophilicity and large dimensions, as well as insulin degradation due to the pH and enzymes in the stomach and small intestine [10,13], leading to low bioavailability [14]. 

To this date, many efforts have been done to try to improve oral administration of insulin, including the use of nanoparticles (NPs) [15,16]. NPs may overcome the mentioned difficulties by protecting the protein drug from the hostile conditions of the gastrointestinal tract (GIT) and improving its absorption [14]. This can be achieved by adjusting the surface charge, shape, size, and hydrophobicity of the NPs, and other characteristics [17]. Furthermore, the use of NPs allows control over the drug release [17,18]. Previous reports have shown that using synthetic and/or natural polymers to nanoencapsulate insulin improves its absorption in the intestine [17,19]. Moreover, by choosing the correct materials to prepare the nanoformulation, certain beneficial characteristics can be achieved. The developed insulin formulation for oral delivery entails insulin-loaded poly (D, L-lactic-co-glycolic acid) (PLGA) NPs coated with chitosan and polyethylene glycol (PEG), with an external coating composed of bovine serum albumin (BSA). PLGA is widely used as a nanocarrier, because it is biodegradable and, when in combination with polyethylene glycol (PEG), allows for longer plasmatic circulation time [20]. The addition of (BSA), as an outer coat of the NPs, acts as a protective layer against proteolytic enzymes of the GIT, allowing insulin to reach systemic circulation intact and increasing its bioavailability at its absorption site [21,22,23], i.e., the intestine. But one of the most important biomolecules in this work comes from the sea. In this case, the ocean has been shown to provide a rich place with great biodiversity and chemical entities with proven bioactivities. Chitosan is generally derived from the shells of shrimp and other sea crustaceans and it acts as a permeability enhancer by opening the tight junctions of the intestinal epithelium, facilitating paracellular and transcellular transport [23,24,25]. Moreover, chitosan prolongs the residence of time of chitosan-coated formulations in mucosae, through its interactions with mucins [26,27].

This work focuses on preparation and characterization of double-coated insulin-loaded NPs by using the following techniques: dynamic light scattering and electrophoretic mobility for size and surface charge analysis, respectively; scanning electron microscopy to assess the NPs’ surface; HPLC for determination of encapsulation efficiency; circular dichroism to evaluate insulin’s activity after the encapsulation and ex-vivo and in silico studies to access the mucoadhesion. Safety assessment was in vitro preliminarily assessed using cells and then by in vivo using animal models. Finally, the efficacy of the formulation was in vivo assessed by evaluating the effect of glycemia in diabetic rats following oral administration of the double-coated insulin-loaded NPs.

## 2. Materials and Methods 

### 2.1. Materials

#### 2.1.1. Chemicals 

Pluronic^®^ F167 (POLX), pepsin (250 IU/mL), BSA (MW 66 kDa) and PEG 4000 were acquired from Sigma-Aldrich (St. Louis, MO, USA). PURASORB^®^ PDLG 5002- PLGA Ratio L/G% 50:50 (MW 45,000–75,000 Da) was purchased from Purac (Gorinchem, The Netherlands). Chitosan from crab shells with low molecular weight (Aldrich), 75–85% deacetylated, was used [27]. The insulin used was Insuman Rapid (Sanofi, Paris, France), a fast-acting insulin, at a concentration of 100 IU/mL. Water MiliQ by Millipore Corporation (Burlington, MA, USA). All the chemical products and solvents used are of analytic purity grade.

#### 2.1.2. Animals

Male Wistar rats 8–10-weeks old, with an average weight of 200 g, were purchased from Charles River (Barcelona, Spain). Males were chosen over female Wistar rats due to the potential influence of female hormones over insulin sensitivity [28]. The animal housing was kept at the controlled temperature of 22.0 ± 1.0 °C, humidity at 50.0 ± 15.0% and a cycle of light of 12 h. Animals were kept under standard hygiene conditions, fed with commercial chow and given acidified drinking water *ad libitum*. The feed was removed 12 h prior to the day of treatment.

All animal experiments (Protocol title: Improvement of insulin oral availability through encapsulation in polyelectrolyte complex nanoparticles, n.° POCI/SAU-FCF//59940) was conducted in accordance with the EU Directive (2010*/*63*/*UE), the Portuguese law (DR 113*/*2013, 2880*/*2015 and 260*/*2016) and the Animal Welfare Commission of the Faculty of Pharmacy, University of Coimbra, approved by Ethics Committee of the Faculty of Pharmacy, University of Coimbra and by the competent national authority Direcção-Geral de Alimentação e Veterinária (DGAV).

### 2.2. Methods

#### 2.2.1. Preparation of NPs

The NPs were prepared according to the modified-spontaneous emulsification solvent diffusion method [29]. An organic solution containing PLGA and insulin was prepared in a non-aqueous solvent mixture. The latter suspension was gradually added to an aqueous solution containing a surfactant at 0.1%, POLX, dissolved in water, at pH 4.5, at room temperature (25 °C) and stirred at 800 rpm (Heidolph MR3001, Heidolph Instruments, Schwabach, Germany), for 15 min [30]. All parameters were considered based on previous studies reported [29,30]. Next, the NPs were coated with a chitosan aqueous solution (0.03% w/v, previously solubilized with glacial acetic acid at 1%) enriched with PEG (0.150%, w/v), by mixing the previous solution with an aqueous solution containing these compounds. This was done at room temperature, and the NPs were constantly stirred at a speed of 180 rpm, for 30 min. Then, the coated NPs were re-coated with a BSA aqueous solution, at a concentration of 1 g/mL, by stirring the chitosan-NPs solutions with this aqueous solution at a speed of 100 rpm, for 30 min. After the final coating, the formulation was centrifuged at 10.000× *g*, for 15 min (Beckman Instruments centrifuge, Inc., Brea, CA, USA), in order to remove all the reagents that did not react.

#### 2.2.2. NPs Characterization

##### Mean Size, Polydispersity Index (PI), and Zeta Potential Analysis

The uncoated NPs, chitosan-coated NPs and double-coated NPs were characterized regarding their mean particle size, polydispersity index (PdI) and surface charge as zeta potential. Particle size and PdI were measured in diluted samples with water MiliQ (1:10, v/v) using Dynamic Light Scattering (Zetasizer Nano S, Malvern Instruments, Malvern, Worcestershire, UK), and performed in triplicates. Zeta potential was analyzed using an electrophoretic mobility assay using the same equipment, using NPs diluted in water MiliQ (same dilution).

##### Surface and Morphological Analysis

The morphology of single-coated and double-coated NPs was observed by Scanning Electron Microscopy (SEM). Aliquots (10 µL) of particle suspensions were scattered over round glass coverslips coated with poly L-lysine, that were previously attached with a double face tape to the microscope stubs. The samples, after dying in a desiccator, were coated with a thin layer of gold and observed on a JEOL 5200 LV scanning electron microscope (JEOL Ltd., Tokyo, Japan) at 20 kV. The images were digitally recorded. 

#### 2.2.3. Determination of Encapsulation Efficiency (EE)

The percentage of insulin encapsulated was determined indirectly by quantifying the amount of insulin present in the supernatant after centrifugation of samples (10.000× *g*, 15 min; using a Beckman Instruments centrifuge, Inc., Brea, CA, USA). This quantification was done by HPLC (Hitachi System LaCrom Elite, Column oven, Diode Array Detector Uv-vis and Pump, Tokyo, Japan), using a Column Waters Symmetry C18, 5 μm 4.6 × 150 mm, with an isocratic flow of 0.7 mL/min. The mobile phase was composed by acetonitrile:TFA water (60:40) (v/v). The measurements were performed in triplicates and the calibration was done with a standardized solution of insulin, at 220 nm wavelength. The linearity range was established in the 1.09–70 μg/mL range and the detection limit was 0.359 μg/mL and the quantification limit was 1.087 μg/mL. The retention time was equal to 3.1 min. Encapsulation efficiency (EE, %) was then determined by using Equation (1):

(1) $$EE\left(\%\right)=\frac{\left(Initially\;added\;insulin-insulin\;present\;in\;supernatant\right)}{Initially\;added\;insulin}\times 100$$

#### 2.2.4. Determination of Recovery Yield (RY)

NPs were recovered after being centrifuged and lyophilized at −49 °C for at least 48 h (Freezone 2.5 L, Freeze-dryer Labconco, Kansas City, MO, USA). Next, NPs were stored at 4 °C, according to previous works [29]. The recovery yield (RY, %) was determined using Equation (2):

(2) $$RY\;\left(\%\right)=\frac{final\;mass\;of\;nanoparticles}{mass\;of\;components\;used\;in\;formulation}\times 100$$

#### 2.2.5. Insulin Activity

##### Circular Dichroism (CD)

The secondary structure of insulin was analyzed by CD spectroscopy. Circular dichroism (CD) spectra were recorded on a JASCO J-720 spectropolarimeter (JASCO, Hiroshima, Japan) with a 180–700 nm photomultiplier (EXEL-308). CD spectra were recorded in the far UV range from 260 to 200 nm with quartz Suprasil^®^ CD cuvettes (0.1 cm). The measurements were done at ~23 °C in a room with controlled temperature. Each CD spectrum is the result of six accumulations recorded in degrees. The following acquisition parameters were used: data pitch, 0.5 nm; bandwidth, 2.0 nm; response, 2 s and scan speed, 50 nm/min. Samples for CD analysis were obtained after disrupting the chitosan-coated insulin-loaded NPs in PBS (USP 30), pH 7.4 and ultrasounds. The insulin concentration of every sample was normalized to 1 mg/mL and compared with equal concentrations of non-encapsulated insulin. 

The CD signal values obtained at 208 nm were used to estimate the α-helical (%) content of the protein [30,31]. CD measurements were expressed as the mean residue ellipticity (MRE in deg cm^2^ dmol^−1^), calculated from Equation (3):

(3) $$MRE=\frac{CD\;\left(mdeg\right)}{Cp\times N\times l}$$

where N is the number of amino acid residues (51 for insulin), l is the length of the optical path (0.1 cm) and C_p_ is the concentration of the protein. The α-helical content (%) is calculated from the MRE values at 208 nm, using Equation (4):

(4) $$\alpha -helix\;\left(\%\right)=-\frac{\left(MRE_{208nm}-4000\right)}{\left(33000-4000\right)}\times 100$$

#### 2.2.6. In Vitro Release Assay

A specific amount (10 mg) of double-coated insulin-loaded NPs were placed in 50 mL HCl (pH 1.2), simulating gastrointestinal conditions, and always respecting sink conditions according to insulin’s solubility. The assay was performed at 37 °C with continuous stirring (100 rpm), in a magnetic multiplate (Heidolph MR3001, Heidolph, Schwabach, Germany), for 2 h. Aliquots (1 mL) were collected at 0.25, 0.5, 1, and 2 h and replaced using fresh medium to have a constant final volume. After this time period, the NPs were centrifuged (× *g*, 10 min) and the pellet was transferred to PBS at pH 6.8. Release assay continued at a speed of 100 rpm, at 37 °C, for 6 h. Aliquots (1 mL) were collected at 0.25, 0.5, 1, 2, 4, and 6 h, when the assay experiment was stopped. At the determined time points, the aliquots were collected and centrifuged (1500× *g*, 10 min), and the pellet was resuspended in the medium solution and returned to the release medium. The aliquots were analyzed and the concentration of insulin was determined by HPLC following the method previously described, in triplicate, and according to Equation (5) [29,31,32]:

(5) $$Released\;Insulin\;\left(\%\right)=\frac{Cn\;V+Vi\;\sum_{i=0}^{\;n-1}Ci\;}{Total\;mass\;of\;the\;particles\;X\;drug\;content\;}\times 100$$

where Cn was insulin concentration at time n (time points), V was total volume of medium, Vi was volume of sample collected at time i, and Ci was concentration of insulin of sample collected at time i (initial time point).

#### 2.2.7. Ex-Vivo Mucoadhesion Study

A TA-XT*Plus* Texture Analyser (Stable Micro Systems, Godalming, UK) equipped with a 5 k load cell was used for mucoadhesion tests [33]. A fresh *Wistar* chemically-induced diabetic rat (explained in section in vivo efficacy assay) small intestine was harvested and opened longitudinally, cleaned and cut into pieces that fit the movable cylindrical probe with the lumen side facing outwards, attached by a double-face tape to the probe. The rat intestine was also placed in a static holder aligned with the probe, also attached by double-face tape. Between the intestine’s portions and in contact with the stationary part, the suspensions with NPs were placed: non-encapsulated insulin, uncoated NPs and double-coated NPs after digestion with pepsin, in equal concentrations of insulin. The double-coated NPs were incubated with pepsin, for 2 h at 37 °C, in order for the albumin to be digested, according to Pharmacopeia USP. The simulated gastric fluid was composed of 3.2 g/L of pepsin (with activity of 800 to 2500 units per mg of protein), sodium chloride, and hydrochloric acid. During the experiment, the movable part was lowered, until coming into contact with the mucosa, up to a force of 20 gf, and then raised at a constant speed of 0.25 mm/s. 

The displacement and the forces of compression and detachment were recorded. A curve of force (gf) versus time (s) was obtained for each experiment and the peak force of displacement (F_max_, gf) and area of the peak (AUC, gf.s) were acquired from this data. The experiments were performed five times for each sample.

#### 2.2.8. In Silico Mucoadhesion Analysis 

For mucoadhesion analysis of chitosan-coated NPs versus uncoated PLGA NPs, energetic and geometric stability of the polymer-mucin molecular complexes were determined using static lattice atomistic simulations (molecular mechanics simulations; Chemlite30, Hypercube Inc., Gainesville, FL, USA). The structures of PLGA and PEG were generated as natural bond angles while the ones of chitosan and glycosylated mucin (MUC) were generated using the saccharide building and sequence editor tools, respectively [34]. The individual molecules (PLGA, PEG, chitosan, and MUC) as well as the molecular complexes (PLGA-MUC and chitosan/PEG-MUC) were energy minimized and optimized using MM+ Force Field algorithm. For geometrical optimization, a Polak–Ribiere Conjugate Gradient method was employed until an RMS gradient of 0.001 kcal/mol was achieved [35].

#### 2.2.9. Preliminary Safety Assessment

##### In Vitro Assessment

The safety of double-coated insulin-loaded NPs was assessed in vitro by a MTT assay performed in Caco2 cells, a human intestinal cell line commonly used for this type of assessment. These cells were kept in Dulbecco’s Modified Eagle’s medium (DMEM) high-glucose (4.5 g/L), supplemented with 10% fetal bovine serum and 100 IU/mL of penicillin and 100 μg/mL streptomycin (hereafter complete medium). Cells were maintained at 37 °C, with a 5% CO_2_ atmosphere, and checked every 2 to 3 days, until a confluence of 80% was reached. Then, the cells were seeded in 96-well plates, at a concentration of 5.0 × 10^4^ cells/mL. The cells were incubated with double-coated insulin-loaded and empty NPs, as well as non-encapsulated insulin. The concentrations tested, for both free and nanoencapsulated insulin, ranged from 0.0625 to 1 IU/mL, and the equivalent was tested for double-coated empty NPs. After 24 h, complete medium was removed, the cells were washed with phosphate buffered saline (PBS) and the MTT solution in incomplete medium (0.5 mg/mL) was added. The cells were incubated for 4 h. After the incubation time, Dimethyl Sulfoxide (DMSO) was added in order to dissolve the formazan crystals. Absorbance was measured at 590 nm using a BioTek ELx800 Absorbance Microplate Reader (BioTek Instruments, Inc., Winooski, VT, USA).

##### In Vivo Preliminary Safety Assessment

The in vivo preliminary safety assessment was performed in 18 male Wistar rats, weighing approximately 200 g. The animals were randomly separated into five groups: The test group (n = 5), to which 50 IU/kg of double-coated insulin-loaded NPs was orally administered; the vehicle control group (n = 5), which received empty double-coated NPs, by oral gavage; the negative control (n = 2), which received PBS orally; the oral insulin control (n = 3), that received 50 IU/kg of commercial insulin not incorporated in NPs, orally; and finally, the control for insulin’s activity (n = 3), to which 4 IU/kg of commercial insulin was subcutaneously administered. After 6 h, urine samples were collected from all animals. The animals were then euthanized, plasma was collected, for hematological and biochemical analyses, and spleen, stomach, liver, intestine and kidney were harvested for histologic analysis. The organs were fixed in 10% formalin and embedded in paraffin. Five micrometer sections of each organ were prepared for hematoxylin-eosin staining. The stained slices were examined under an Olympus BX51 microscope (Olympus Corporation, Tokyo, Japan) and images were captured with NanoZoomer-SQ Digital slide scanner (Hamamatsu Photonics, Hamamatsu City, Japan). The urine samples collected were tested for leukocytes, urobilinogen, bilirubin, hematuria, nitrites, pH, density, proteinuria, glycosuria, and ketonic bodies, using Uritest 10 V Urinalysis Reagent Strips. Plasma samples were tested to quantify IL-6, ALT (to determine liver toxicity), creatine and urea (to determine kidney toxicity). 

#### 2.2.10. In Vivo Efficacy Assay

##### Diabetes Mellitus Induction

In order to chemically induce diabetes mellitus in the male Wistar rats, streptozotocin (STZ), prepared in citrate buffer 0.1 M at pH 4.5 (UPS 30), was administered intraperitoneally (i.p., 65 mg/kg of body weight). After this procedure, animals were exposed to a 5% glucose solution during the night, in order to avoid reactional hypoglycemia, caused by the STZ. The animals were classified as diabetic when glycemia was higher than 300 mg/dL, measured on the third day post-administration [31,36]. The experimental protocol was started 10 days after STZ administration [29,36].

##### Study Design

For the in vivo efficiency assay (n = 14), animals were randomly divided into three groups: The test group (n = 5), in which 50 IU/kg of NPs formulation was orally administered; the negative control group (n = 3), to which empty NPs were administered; in the third group, insulin was administered orally (n = 6). The formulation efficacy is translated by decreasing glycemia, glycemia was measured at the following time points: 30 min, 1, 2, 4, 6, and 8 h. Glycaemia levels were determined by measuring glucose oxidase/peroxidase, using a glucosometer (OneTouch^®^ Verio^®^IQ, Milpitas, CA, USA). The animals were then sacrificed. The plasma to which the double-coated insulin-loaded NPs were administered was collected and its insulinaemia was determined by electrochemiluminescence immunoassay/(Roche-Cobas^®^).

#### 2.2.11. Statistical Analysis

Each value is presented with a mean value ± SD. The statistical differences were evaluated with t-Student test and ANOVA. These tests allow to compare two or multiple groups, respectively. All analyses were conducted in GraphPad Prism Version 5.03 (GraphPad Software, San Diego, California, USA) and the differences were deemed significate at a *p* < 0.05.

## 3. Results

### 3.1. NPs Characterization: Size, Surface Charge, PI, Morphology, EE, and RY

**Figure 1:**
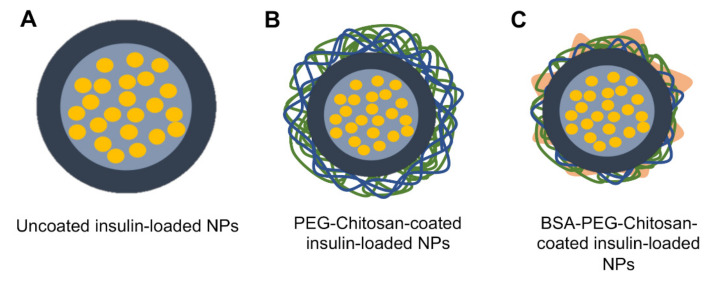
Double-coated insulin-loaded nanoparticles (NPs) throughout the formulation and coating processes: (**A**) uncoated insulin-loaded poly (D, L-lactic-co-glycolic acid) (PLGA) NPs; (**B**) chitosan-coated insulin-loaded PLGA NPs; and (**C**) double-coated insulin-loaded PLGA NPs.

Mean size and PdI are shown in Table 1. Insulin-loaded NPs have a larger mean particle size than empty NPs. It is assumed that the increase in particle size was related to the encapsulation of insulin. Besides the particle size, there are other important parameters that contribute to an increase absorption of the NPs in the intestinal mucosa, such as NPs charge. This property was evaluated by measuring the NPs zeta potential in all phases of production, also shown in Table 1. A representative scheme of insulin NPs is displayed in Figure 1. The uncoated empty and uncoated insulin-loaded NPs (Figure 1A) showed a negative surface charge. This value was coherent with the PLGA charge at the considered pH, as previously reported [37]. The NPs charge was inverted to positive, in both empty NPs and insulin-loaded NPs by coating the NPs with chitosan, a cationic polymer, and PEG (Figure 1B). The final formulation was obtained after the BSA coating. The charge of the NPs coated with BSA, both empty and insulin-loaded double-coated NPs (Figure 1C), remained positive, although slightly less positive. These results were as expected and coherent with what was described in previous studies [23,38].

**Figure 2:**
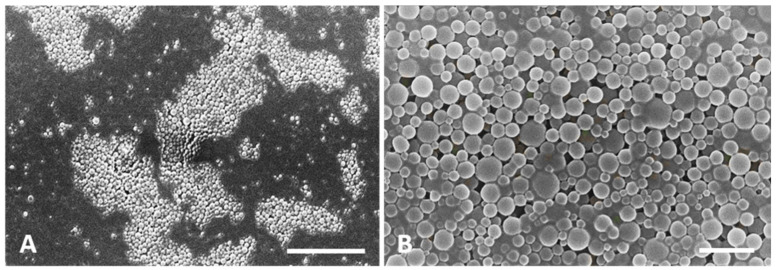
SEM micrographs of double-coated NPs. (**A**) Empty NPs. (**B**) Insulin-loaded NPs. Note in both cases the well-defined NPs spherical shape. Scale bars = 5 µm.

Figure 2 shows the images obtained by scanning electron microscopy for both double-coated empty and insulin-loaded NPs, where NPs seem to have a well-defined spherical shape.

The EE was 59.8 ± 2.6%, of the insulin used in the initial formulation. The RY was 80.6 ± 1.1%. 

### 3.2. Insulin Secondary Structure

**Figure 3:**
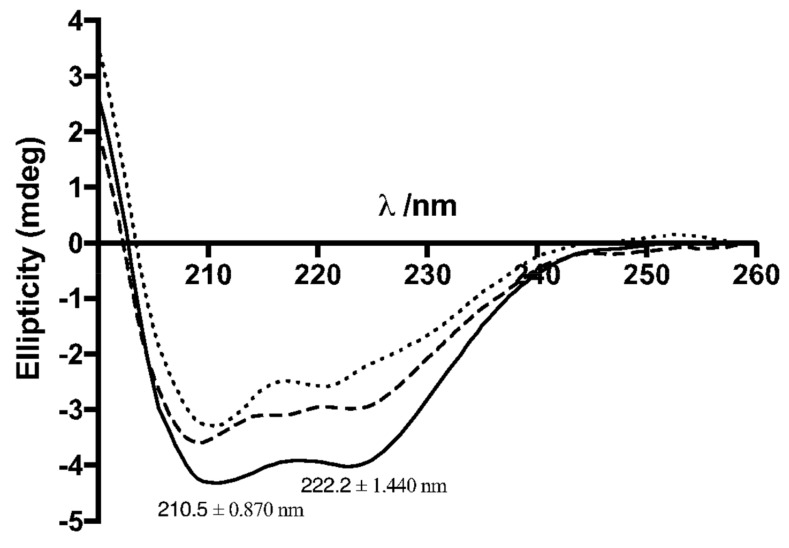
Circular dichroism (CD) spectra of non-encapsulated insulin (**^___^**), uncoated NPs (---) and chitosan-coated NPs (^…^) in the Far UV range.

Circular dichroism (CD) was used to evaluate the secondary structure of nanoencapsulated insulin, after being released, which was shown to remain intact, as illustrated in Figure 3, where it is observed the characteristic spectra of a protein with an alpha helix structure. Figure 3 presents CD spectra showing two peaks, which are characteristic of insulin’s CD spectrum, at 211 nm and 222 nm. An estimate of the α-helical content was done using the MRE at 208 nm. This yielded the following values 31%, 28%, and 27% for non-encapsulated insulin, uncoated NPs and chitosan-coated NPs, respectively. Thus, only a small decrease in the α-helical content was detected along the preparation and coating of NPs, suggesting no insulin fibrillation/aggregation among the conditions tested. Moreover, these findings were also supported by HPLC (observing the same retention time of insulin in all samples) and then after in vivo oral administration (Section 3.7).

### 3.3. In Vitro Release Assay

**Figure 4:**
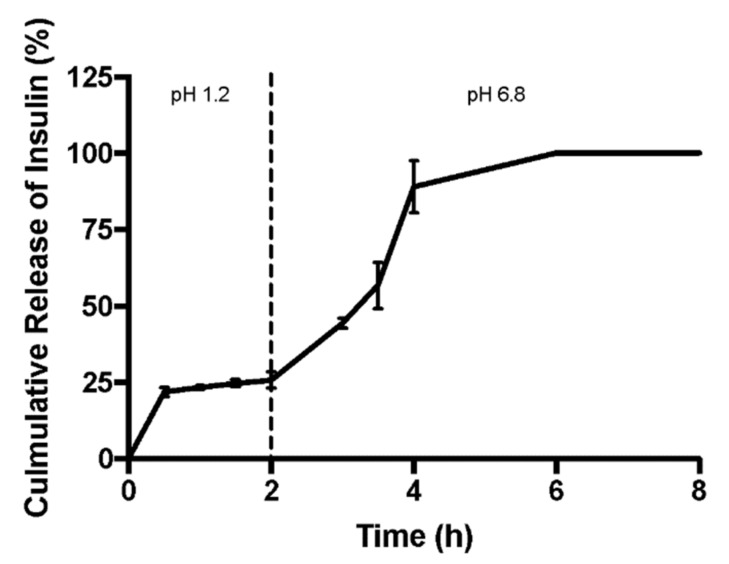
Release assay for the double-coated insulin-loaded NPs in acidic medium until 2 h and conducted in neutral medium from 2 to 8 h, mimicking the pH range of the gastrointestinal tract (GIT).

This assay was performed in order to better understand insulin’s release profile in the gastrointestinal tract, and the results are shown in Figure 4. Temperature was kept at 37 °C and stirring was always constant. Firstly, the nanoencapsulated insulin was kept in acidic medium and aliquots were collected up to 2 h. In the acidic medium simulating gastric conditions, chitosan is probably starting to be dissolved at acidic pH and insulin release was 25.6 ± 3.4% after 2 h. For the neutral medium (mimicking the intestine), NPs are less coated and insulin immediately started to release and it was 100% released after 4 h. 

### 3.4. Mucoadhesion Study

#### 3.4.1. Ex-Vivo Mucoadhesion Study

**Figure 5:**
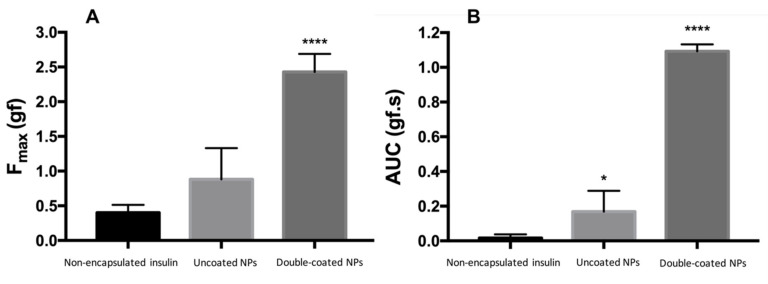
The peak force of displacement (F_max_, gf) (**A**) and the area of the peak (AUC, gf.s) (**B**) obtained from the force versus time curves. The results are presented regarding the mean value ± respective SD (* *p* < 0.0332; **** *p* < 0.0001).

The maximum peak force of displacement (F_max_, gf) and area of the peak (AUC, gf.s) were obtained. The F_max_ correlated well with AUC (r^2^ = 0.99). The results are shown in Figure 5. Pepsin digested double-coated NPs were shown to be more mucoadhesive than non-encapsulated insulin and uncoated NPs, having the highest value for both F_max_ and AUC. Non-encapsulated insulin solution was found to promote the least mucoadhesive effect of all, as expected. 

#### 3.4.2. In Silico Mucoadhesion Analysis

**Figure 6:**
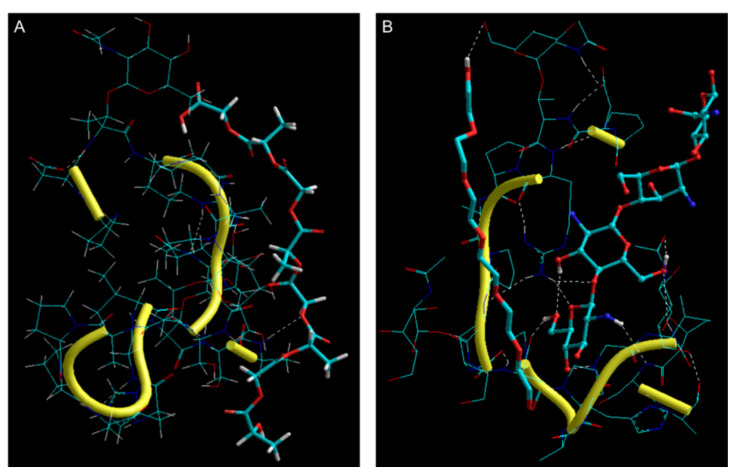
Representation of the geometrical preferences of (**A**) PLGA-MUC; (**B**) Chitosan/PEG-MUC after molecular mechanics simulations in vacuum [Colour code for elements: C = cyan; H = white; O = red; N = blue]. PLGA: tube rendering; MUC: stick rendering and yellow second.

Table 2 and Figure 6A represent the geometrical preferences of NPs core and NPs coating with mucin (MUC) after molecular simulations, in vacuum, whereas Table 3 and Figure 6B represent the molecular, energy, and geometrical attributes inherent to PLGA-glycosylated MUC and chitosan/PEG-MUC molecular complexes, respectively. Both the polymer combinations—PLGA and chitosan/PEG—formed molecular complexes with MUC with –ve energy of stabilization hence confirming the polymer selection and application for the intended oral delivery. However, in case of PLGA-MUC molecular complex, all bonding energy terms (bond, angle, and dihedral energies) and two non-bonding energy contributions (H-bonding and electrostatic energy) were destabilized (Table 2). Although the PLGA chain showed good geometrical fit (ΔE_vdW_ ≈ −33 kcal/mol); the torsional strains experienced by the polymer chain (ΔE_angle_ ≈ +18 kcal/mol) retained the polymeric network out of the mucin matrix. Additionally, the PLGA chain not only showed partial intermolecular H-bond formation (…C-O…OH-C…) with MUC but also reduced the extent of intramolecular H-bonding within the MUC matrix (Figure 6A). With chitosan/PEG-MUC, the energy of stabilization was significantly higher (ΔE ≈ −54 kcal/mol) than PLGA-MUC with all component energies stabilized except angle and H-bonding contributions (Table 3). It is worth noting that the bond angle contribution energy in chitosan/PEG-MUC was much lower than PLGA-MUC as well as the bond stretching and torsional energies in chitosan/PEG-MUC showed favorable stabilization. In addition to this, stabilization of the van der Waals function (ΔE ≈ −24 kcal/mol) led to a close fit of the chitosan chains within the MUC matrix. The close geometrical fit so formed further induced electrostatic interactions (ΔE ≈ −35 kcal/mol) leading to formation of several intermolecular H-bonds such as …C-O-C…H-N…, …N-H…H-N…, and …O-H…H-O… and even intramolecular H-bonds within the MUC matrix (Figure 6B). The PEG chain also contributed to H-bonding with …C-OH…HO-C… interactions with the end group. Although the H-bonding function was partially destabilized (ΔE_angle_ ≈ +0.322 kcal/mol), which may be due to amphiphilic nature of the PEG chain, the overall non-bonding interactions were counter-stabilized by the van der Waals and induced electrostatic interactions. 

### 3.5. Preliminary Safety Assessment of Double-Coated Insulin-Loaded NPs

#### In Vitro Preliminary Safety Assessment (MTT)

**Figure 7:**
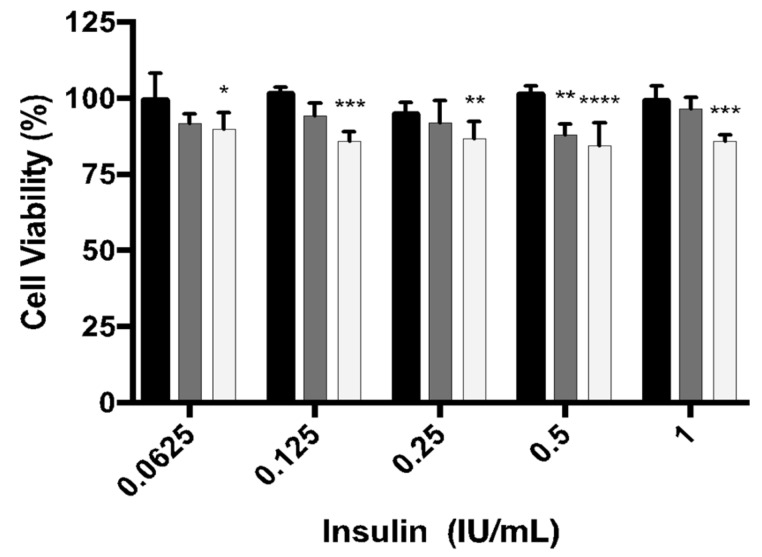
Cell viability (%) of cells treated with double-coated insulin-loaded NPs (empty columns), double-coated empty NPs (grey columns) and non-encapsulated insulin (full columns). The NPs concentrations tested ranged from 0.0625 to 1 IU/mL. The results were presented regarding the mean value ± SD (* *p* < 0.0332; ** *p* < 0.0021; *** *p* < 0.0002; **** *p* < 0.0001).

In order to preliminarily evaluate the safety of the double-coated insulin-loaded NPs, its cytotoxicity activity was assessed by performing a MTT assay. MTT is commonly technique to access the mitochondrial activity [39]. Caco2 cells were incubated with the double-coated insulin-loaded and empty NPs, as well as non-encapsulated insulin, and the results, presented as % of cell viability, are shown in Figure 7. Double-coated empty NPs led to a slight decrease in cell viability, although the lowest mean value was around 85%, at a concentration of 0.5 IU/mL. Looking at the results regarding the double-coated insulin-loaded NPs, we can see the same tendency, with a mean value of around 87% of viable cells.

### 3.6. In Vivo Preliminary Safety Assessment

Animals’ behavior remained unchanged, showing no signs of distress. Different parameters were evaluated in the urine (i.e., leucocytes, urobilinogen, bilirubin, hematuria, nitrite, pH, density, proteinuria, glycosuria and ketonic bodies), and the results are shown in Table 4. Looking at the results, it is to note that, apart from hematuria, proteinuria and ketonic bodies, none of the parameters have changed, when compared to the negative control (PBS) group. Both control and test groups had positive cases of proteinuria which might be attributable to the NPs formulation. Although some rats from the insulin-loaded NPs test group showed an increase in proteinuria, it was never higher than 15 mg/mL, which is still very low and thus, deemed an acceptable proteinuria. Test group did not present ketonic bodies. In the group that orally received empty NPs, an increase hematuria was also observed in some animals, but its hematuria was lower than the one observed for the control group.

**Figure 8:**
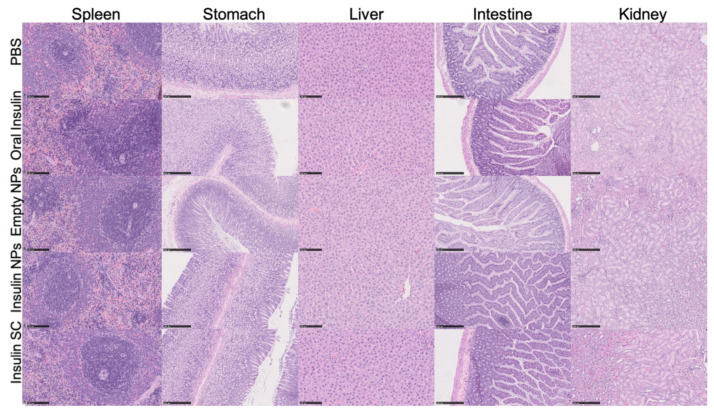
Histological images (spleen and liver with 200× microscopic approach; stomach, intestine and kidney with 100× microscopic approach) of the organs removed for analysis after necropsy (i.e., spleen, stomach, liver, intestine, and kidney). All images are representative of the harvested organs, which showed no histologic alterations (H&E staining).

Figure 8 shows representative images of the organs collected for histological analysis. All organs showcased their physiological traits, without pathological alterations. Thus, the double-coated insulin-loaded NPs did not cause acute organ toxicity to the different organs tested (i.e., spleen, stomach, liver, intestine, and kidney), deeming the formulation as being safe for oral administration.

Table 5 summarizes the main results of the biochemical analysis conducted to quantify the ALT, creatinine, urea and IL-6, serum levels of the rats in the different groups, evaluating general and organ-specific (kidney and liver) toxicity. By comparing the results of the group treated with double-coated insulin NPs to the negative control (PBS), no toxicity was seen. Although ALT was slightly increased, it was still between the reference interval for male Wistar rats (24–29 IU/L [40]), and all the other parameters did not change when compared with the control. Thus, the double-coated insulin-loaded NPs did not cause any acute toxicity, either general or organ-specific, neither inflammatory response, thus reinforcing the safety of the formulation for oral delivery.

### 3.7. In Vivo Efficacy Assay

**Figure 9:**
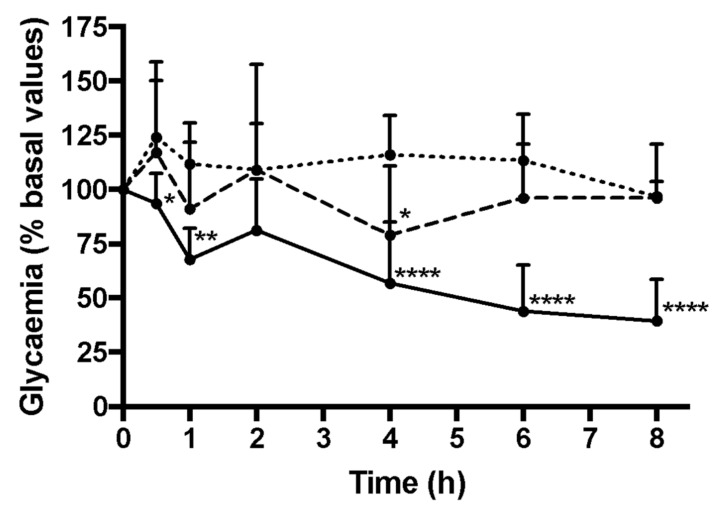
Glycaemia levels of diabetic rats treated with commercialized insulin (…, n = 6, 50 IU/kg), empty (---, n = 3, equivalent amount) and double-coated insulin-loaded NPs (___, n = 5, 50 IU/Kg), orally administered. The results are presented regarding their mean ± SD (* *p* < 0.0332; ** *p* < 0.0021; *** *p* < 0.0002; **** *p* <0.0001).

In order to test the treatment potential of this double-coated insulin-loaded NPs *in vivo*, the formulation was orally administered to diabetic Wistar rats. As insulin’s efficacy is translated by its ability to decrease glycemia values, the glycemia was recorded and the results are shown in Figure 9. It was observed that the diabetic rats treated with double-coated insulin-loaded NPs had a significant decrease in glycemia throughout all time points, in comparison to orally non-encapsulated insulin. The nanoencapsulated insulin led to a sharp decrease in glycemia after 2 h, being that the glycemia of the diabetic rats decreased by between the 4th and 6th hour. After the final time point, animals were sacrificed and the test group’s insulinemia was measured, having a mean insulinemia (human insulin, i.e., our exogenous insulin) of 0.6 μIU/mL, significantly higher than the control group (as expected, there was no increase in plasma insulin in the control group).

## 4. Discussion

Insulin-loaded NPs were successfully prepared and characterized regarding its mean size, PdI and surface charge, morphology and delivery efficiency. The formulated double-coated insulin-loaded NPs had a mean diameter smaller than 600 nm. It is well known that the overall surface area of the particles increases by several orders of magnitude when the particle size is on the order of the nanometer. However, the ideal particle size for the NPs to be absorbed by the intestinal mucosa is still not consensual. Some studies have shown that the particle size should be smaller than 5000 nm in order to be absorbed in the intestinal mucosa [39], whilst other studies states the limit should be between 130 and 950 nm or, in order to optimize NPs uptake by M cells, a mean size less than 500 nm [41]. Altogether, the previous studies presented lead us to believe that the prepared formulation will be uptaken by M cells in the Peyers patches [41], as the obtained particles are of a smaller size than the size limits mentioned. Furthermore, during the formulation process an accentuated decrease in size was seen when the BSA coating was added. This is due to the BSA coating causing a closure of the void spaces present throughout the NP, compacting it, and was also seen in other studies using similar coatings [22]. Nonetheless, the size of the double-coated insulin-loaded NPs will decrease throughout its path in the GI tract and after being absorbed, due to digestion of the BSA coating and loss of chitosan coating. On the other hand, NPs surface charge is also very detrimental for particle absorption in the intestine mucosa, as positively-charged particles are more adherent to the intestine mucosa [41]. In general, mucus is mainly composed of water (~95% w/w), mucins (~0.2% to 5.0% w/v), salts (~0.5% to 1.0% w/w), globular proteins (~0.5% w/v), lipids (1%–2% w/w), DNA, cells and cellular debris [42]. The high content in sialic acid and sulphate in most mucin glycoproteins confer a strongly net-negative surface charge [42]. NPs surface charge was accessed by measuring the zeta potential of empty NPs and insulin-loaded NPs, throughout the addition of the different coatings. The results shown in Table 1 indicate that the PLGA NPs were successfully coated, as we can see the change in zeta potential throughout the application of the different coatings. Chitosan was successful in inverting the NPs zeta potential from negative (uncoated) to positive. Although the final charge, when the BSA coating is added to the NPs, is slightly less positive than when the PLGA NPs are coated with chitosan and PEG, when the particles reach the intestine mucosa it is expected that the BSA coating will no longer be present as previously described [22,23]. Thus, when the NPs reach this region its surface charge will be positive, which is intended, as positively-charged particles are more adherent to the intestine mucosa, by interactions with mucins, negatively-charged glycosylated proteins [10], and, consequently, it might increase the residence time of the NPs in the mucosa, enhancing the bioavailability of the insulin [22,23].

NPs morphology was assessed by SEM and a well-defined spherical shape of the formulated NPs, characteristic of the used polymers, was observed [25]. The spherical shape of the NPs is important as it allows a greater contact surface with the intestine mucosa, facilitating the uptake of the NPs [43]. Previous studies have already taken advantage of NPs spherical shape to achieve higher absorption of orally insulin [29,43,44].

Insulin-loaded PLGA NPs were successfully prepared and coated with chitosan-PEG and BSA. The obtained encapsulation efficiency was of 60%. Although a higher value would be preferable, some steps during the formulation process might have caused lower encapsulation efficiency. The obtained value might be due to the pH oscillations, which has been proven to influence encapsulation efficiency [29], caused by the addition of the different coatings. Insulin has an isoelectric point of 5.3 and therefore it is positively charged at pH 4.5, which might lead to interactions between the different materials, namely BSA, chitosan, PLGA and insulin itself, which can cause some diffusion of insulin from inside to outside of NPs [36,45]. Furthermore, insulin is hydrophilic, thus it has higher affinity to the aqueous (external) phase, which increases the odds of it diffusing out of the polymeric matrix. Finally, the stirring rate used during the addition of the coatings might have also caused some insulin loss, although without change in its biological activity. Nevertheless, a recovery yield of 81% was obtained, which will undoubtedly have a good impact at industrial scale level.

Post-encapsulation insulin was accessed by CD analysis to evaluate insulin’s secondary structure as for protein drugs to be biologically efficient, their structural integrity must be kept. As Brange et al. stated, insulin’s secondary structure is characteristically composed by alpha helix motifs and the maintenance of the structure stability allows insulin to perform its pharmacological activity [46]. Analyzing the results of the CD shown in Figure 3, we can observe very close CD spectra, suggesting that insulin’s secondary structure was preserved during or after the encapsulation process. Although there are other techniques available for the assessment of proteins’ secondary structure, CD is a very suitable technique and commonly used due to being a faster technique and requiring a smaller sample [47,48,49]. Regardless of CD results deeming post-encapsulation insulin as being active, its activity was also confirmed in vivo, by analyzing the decrease of glycemia in diabetic rats after oral administration of the double-coated insulin-loaded NPs. Lastly, the coating with BSA was effective in maintaining insulin’s activity after oral dosing like our group had already demonstrated [22,30]. The presence of active insulin was also confirmed by measuring the insulinemia in diabetic animals to which double-coated insulin-loaded NPs was administered, supporting the presence of human insulin in its blood flow.

PLGA is a FDA approved hydrophobic polymer that allows total release of insulin after administration, due to insulin’s hydrophilic nature, and is easily degradable by endocytosis [7,12,50,51]. The degradation products of PLGA are also easily included in Krebs cycle [52]. However, due to their negative charge, insulin-loaded PLGA NPs have poor adhesion to the intestinal wall. This fact led us to an additional coating of our NPs with a polycationic polymer, such as chitosan. With chitosan, we will expect that mucoadhesion in the intestine mucosa increases, contributing to an increase in the permanence period of the insulin in the mucosa [7,12], facilitating its passage through the epithelial cellular junctions [53]. This fact was confirmed by ex-vivo assessment where chitosan-coated NPs have the highest values for both F_max_ and AUC. Simulations of molecular interaction in silico conducted in vacuum provided interesting corroboration with the ex-vivo findings. Although both the polymer combinations, PLGA and chitosan/PEG, formed molecular complexes with MUC, the energy of stabilization was significantly higher for chitosan/PEG-MUC (ΔE ≈ −54 kcal/mol) than for PLGA-MUC (ΔE ≈ −7.5 kcal/mol). These results confirm the preferable mucoadhesive profile of chitosan/PEG-coated NPs as compared to the uncoated PLGA NPs, as predicted in the ex-vivo results.

The development of suitable and biocompatible drug delivery systems is a prerequisite, especially for a chronic disease like diabetes [17]. MTT assay was done to assess cytotoxicity and exhibited good results as it demonstrated that double-coated insulin-loaded NPs did not alter cell viability. Regarding empty NPs, the lowest mean value of viability found was 85%. This value of cell viability of the empty NPs could be attributed to the NPs’ structure by itself as previously demonstrated [54], and it is not noticeable in insulin-loaded NPs possibly due to the insulin’s presence, since insulin can act as a growth factor, promoting cell growth.

In vitro assays are extremely useful tools, but they cannot accurately predict the in vivo behavior of all formulations. Therefore, an in vivo preliminary safety assessment study was also performed. To assess NPs formulation safety when orally administered, animals’ behavior was closely monitored during the test. No death occurred after the treatment. When comparing the urine tests results, biochemical analysis and histological images of insulin-loaded NPs and negative control group (PBS group), it is displayed that there was no obvious damage to animals. Thus, in vivo studies results were in agreement with in vitro MTT assay, i.e., our insulin-loaded NPs are safe for oral administration.

The presented research entails to develop an insulin formulation suitable for oral delivery as an alternative treatment for the commercially available subcutaneous insulin. Oral administration is considered as the best route of administration because of its cost-effectiveness and well-established acceptability. In addition, it allows avoiding the use of injections. An additional advantage is related to a more physiological action by its direct effect on hepatic glucose production. If the insulin would be absorbed in the gut, insulin would be transferred directly toward the liver. At the liver, the exogenously insulin would control hepatic glucose production to the same extent similarly as this is induced by endogenously insulin in healthy subjects. This more physiological delivery would be associated with reduced peripheral hyperinsulinemia in contrast to SC administration. In terms of pharmacokinetics, as representative example, in a small study made by Cernea et al. 2004 [55], male subjects under euglycemic conditions, oral insulin spray was associated with a higher Cmax, shorter T_max_, and faster time to peak glucose uptake compared with SC insulin. The short T_max_ and the 120-min duration of effect of oral insulin spray suggest it may be a promising alternative for fulfilling meal-related insulin requirements in persons with diabetes. Another encouraging study was done with a few number of subjects with 8 mg of oral insulin formulation [56]. Administration of an oral form of insulin in the fasted state demonstrated a significant effect on insulin absorption. This substantial effect was seen by a reduction of blood glucose (7%–37%), decline in C-peptide levels (13%–87%), as well as an elevation of insulin level (20%–120%). It was noticed that some subjects developed symptomatic hypoglycemia. Insulin formulations were well tolerated. No adverse or serious adverse events have been reported. Therefore, we aimed to develop a new oral insulin formation and to evaluate if the developed formulation is effective when administered orally. The results showed that the formulation was effective in reducing glucose levels of diabetic rats (Figure 9). A previous study comparing the subcutaneous administration of 4 IU/kg insulin with oral administration of 50 IU/kg of nanoencapsulated insulin coated with chitosan and albumin in Wistar diabetic rats has shown that the NPs formulation achieved a reduction in glycemia of 28% between the 2nd and 4th hour, and 48% between the 8th and 12th hour [29]. When compared to other studied formulations, the formulation reported herein has shown preferable characteristics. Many of the previously developed formulations for oral delivery of insulin have negative surface charge [50,51,52,53,54], not favoring the NPs’ interactions with mucins, and thus not promoting adherence to the intestine’s mucosa [10,22,23]. Besides having a positive surface charge, the double-coated insulin-loaded NPs showed to interact with mucin, therefore proving its mucoadhesive properties, as observed both by the *in silico* simulations of molecular interaction conducted in vacuum, and by the ex vivo experiments. The double-coated formulation also showed lower insulin release in gastric-like conditions after 2 h (22.6%) than other formulations intended for oral delivery of insulin with half and more than half of the encapsulated insulin being released in stomach-like conditions after two hours (~50% [57], ~60% [58]), suggesting that, unlike other formulations, our double-coated insulin-loaded NPs formulation has the potential to deliver insulin in the proper target—the gut. Moreover, our formulation was more efficient in lowering diabetic-rats than others: while our double-coated insulin loaded NPs induced a 50% glycemia decrease 8 h post-oral administration, other studied formulations reported smaller and later hypoglycemic effects, to less than 60% after 12 h [59] or 24 h [60] post-oral administration. Furthermore, when compared to other chitosan-based insulin-loaded NPs, the developed formulation had higher encapsulated efficiency, was more resistant to gastric-like conditions or/and achieved higher decrease in glycemia, at 4 h, than what was seen for other chitosan-based formulations of insulin nanoparticles [61,62,63].

## 5. Conclusions

Oral insulin replacement therapy remains a very appealing alternative to subcutaneous injections for patients with diabetes mellitus. However, it seems that the search for an acceptable insulin oral formulation is much more difficult than initially thought. After decades of failed attempts to produce an oral insulin formulation, the number of published clinical trial reports so far is limited. The hope clearly is to see more clinical data. A suitable drug carrier is important to ensure site-specific sustained drug delivery.

As described herein, our double-coated insulin-loaded NPs formulation showed to be efficient in reducing the glycemia up to 50% in chemically-induced diabetic rats and it is safe. Additionally, the encapsulation was effective and the method of encapsulation did not alter the insulin’s secondary structure. So, it is expected that the drug maintains its integrity when it reaches its biological target. Furthermore, it was proved in the in vitro assays that by encapsulating insulin and coating the NPs with two different layers, insulin was protected from the hostile environment of the GIT. Moreover, the ex-vivo study showed that the chitosan coating exerts its mucoadhesiveness, correlating with the in silico analysis.

Thus, these double-coated insulin-loaded NPs might provide a more efficient and safer platform for delivering insulin by mimicking physiologic processes and directly deliver insulin to the liver rather than via the bloodstream. A great amount of work still remains to be done, but like many other examples in other therapeutic areas, nanomedicine brings new hope to succeed in obtaining an oral treatment of insulin available to diabetic patients.

## Figures and Tables

**Table 1:** Mean size, PdI and zeta potential throughout the different steps in formulation, for both empty and insulin-loaded NPs. All data is presented as mean ± SD (n = 3).

Sample		Mean Size (nm)	PdI	Mean Zeta Potential (mV)
Empty NPs	Uncoated NPs	240 ± 2	0.110 ± 0.016	−39 ± 6
	Chitosan-coated NPs	328 ± 2	0.231 ± 0.015	+48 ± 8
	Double-coated NPs	233 ± 2	0.184 ± 0.016	+34 ± 8
Insulin-loaded NPs	Uncoated NPs	936 ± 4	0.449 ± 0.023	−49 ± 5
	Chitosan-coated NPs	819 ± 7	0.527 ± 0.028	+41 ± 13
	Double-coated NPs	560 ± 9	0.546 ± 0.030	+31 ± 3

**Table 2:** Inherent energy attributes of poly (D, L-lactic-co-glycolic acid)–mucin (PLGA–MUC) conjugate calculated using static lattice atomistic simulations in vacuum.

Energy	MUC	PLGA	PLGA–MUC	ΔE ^a^	
				Structure Stabilizing	Structure Destabilizing
Total ^b^	−166.812	2.638	−171.699	−7.53	
Bond ^c^	5.474	0.288	6.149		0.39
Angle ^d^	70.351	2.534	90.682		17.80
Dihed ^e^	55.173	0.885	59.13		3.07
vdW ^f^	−29.066	−1.062	−62.948	−32.82	
H-bond ^g^	−7.096	−0.007	−6.724		0.38
Elec ^h^	−261.649	0.000	−257.987		3.66

^a^ ΔE_(A/B)_ = E_(A/B)_ – [E_(A)_ + E_(B)_]; ^b^ Total steric energy for an optimized structure; ^c^ Bond stretching contributions; ^d^ Bond angle contributions; ^e^ Torsional contribution arising from deviations from optimum dihedral angles; ^f^ Van der Waals interactions; ^g^ Hydrogen-bond energy function; ^h^ Electrostatic interactions.

**Table 3:** Inherent energy attributes of chitosan/PEG-MUC conjugate calculated using static lattice atomistic simulations in vacuum.

Energy	MUC	Chitosan	PEG	Chitosan/PEG-MUC	ΔE ^a^	
					Structure Stabilizing	Structure Destabilizing
Total ^b^	−166.812	8.822	10.127	−201.742	−53.879	
Bond ^c^	5.474	1.077	0.158	6.706	−0.003	
Angle ^d^	70.351	6.058	0.703	82.729		5.617
Dihed ^e^	55.173	8.755	6.001	66.450	−3.479	
vdW ^f^	−29.066	5.920	3.264	−43.716	−23.834	
H-bond ^g^	−7.096	0.000	0.000	−6.774		0.322
Elec ^h^	−261.649	−12.988	0.000	−307.137	−32.5	

^a^ ΔE_(A/B)_ = E_(A/B)_ − [E_(A)_ + E_(B)_]; ^b^ Total steric energy for an optimized structure; ^c^ Bond stretching contributions; ^d^ Bond angle contributions; ^e^ Torsional contribution arising from deviations from optimum dihedral angles; ^f^ van der Waals interactions; ^g^ Hydrogen-bond energy function; ^h^ Electrostatic interactions.

**Table 4:** Status of the different parameters evaluated in the urine of the rats of the different groups.

	Insulin-Loaded NPs (n = 5)	Empty NPs (n = 5)	Non-Encapsulated insulin, p.o. (n = 3)	PBS (n = 2)
Leucocytes	-	-	-	-
Urobilinogen	N	N	N	N
Bilirubin	-	-	-	-
Hematuria	+/-	+/-	-	+/-
Nitrite	-	-	-	-
pH	5.4	5.9	5.3	5.5
Density	1.03	1.02	1.03	1.03
Proteinuria	+/-	+/-	+/-	+/-
Glycosuria	-	-	-	-
Ketonic bodies	-	-	+/-	+/-

+, one positive case; −, negative result; N, no change.

**Table 5:** Serum levels of ALT, creatinine, urea, and IL-6, of the rats of the different groups. The results were presented regarding the mean ± SD.

Formulation and Number of Animals	ALT (U/L)	Creatinine (mg/dL)	Urea (mg/dL)	IL-6 (ng/dL)
PBS (n = 2)	30.5 ± 12.0	0.39 ± 0.03	37.0 ± 9.9	< 1.5
Oral non-encapsulated Insulin (n = 3)	34. 7 ± 3.8	0.38 ± 0.02	37. 7 ± 6.5	< 1.5
SC non-encapsulated Insulin (n = 2)	21.0± 4.2	0.37± 0.01	41.0± 1. 8	< 1.5
Double-coated Insulin-loaded NPs (n = 5)	34.6 ± 12.1	0.40 ± 0.02	37.4 ± 2.8	< 1.5
Empty NPs (n = 5)	35.6 ± 14.2	0.35 ± 0.06	37.2 ± 1.9	< 1.5
